# Association of Organophosphate Pesticide Exposure and Paraoxonase with Birth Outcome in Mexican-American Women

**DOI:** 10.1371/journal.pone.0023923

**Published:** 2011-08-31

**Authors:** Kim G. Harley, Karen Huen, Raul Aguilar Schall, Nina T. Holland, Asa Bradman, Dana Boyd Barr, Brenda Eskenazi

**Affiliations:** 1 Center for Environmental Research and Children's Health, School of Public Health, University of California, Berkeley, California, United States of America; 2 Rollins School of Public Health, Emory University, Atlanta, Georgia, United States of America; Indiana University, United States of America

## Abstract

**Background:**

Epidemiologic studies suggest that maternal organophosphorus (OP) pesticide exposure is associated with poorer fetal growth, but findings are inconsistent. We explored whether paraoxonase (PON1), a key enzyme involved in detoxification of OPs, could be an effect modifier in this association.

**Methods:**

The study population included 470 pregnant women enrolled in the CHAMACOS Study, a longitudinal cohort study of mothers and children living in an agricultural region of California. We analyzed urine samples collected from mothers twice during pregnancy for dialkyl phosphate (DAP) metabolites of OP pesticides. We analyzed maternal and fetal (cord) blood samples for PON1 genotype (*PON1_192_* and *PON1_−108_*) and enzyme activity (paraoxonase and arylesterase). Infant birth weight, head circumference, and gestational age were obtained from medical records.

**Results:**

Infants' PON1 genotype and activity were associated with birth outcome, but mothers' were not. Infants with the susceptible *PON1_−108TT_* genotype had shorter gestational age (β = −0.5 weeks, 95% Confidence Interval (CI): −0.9, 0.0) and smaller head circumference (β = −0.4 cm, 95% CI: −0.7, 0.0) than those with the *PON1_−108CC_* genotype. Infants' arylesterase and paraoxonase activity were positively associated with gestational age. There was some evidence of effect modification with DAPs: maternal DAP concentrations were associated with shorter gestational age only among infants of the susceptible *PON1_−108TT_* genotype (p-value_interaction_ = 0.09). However, maternal DAP concentrations were associated with larger birth weight (p-value_interaction_ = 0.06) and head circumference (p-value_interaction_<0.01) in infants with non-susceptible genotypes.

**Conclusions:**

Infants whose PON1 genotype and enzyme activity levels suggested that they might be more susceptible to the effects of OP pesticide exposure had decreased fetal growth and length of gestation. PON1 may be another factor contributing to preterm or low birth weight birth.

## Introduction

Organophosphorus (OP) pesticides are a class of widely used, neurotoxic insecticides that include compounds such as chlorpyrifos, diazinon, and malathion. Chlorpyrifos and diazinon were used in household pest control products until 2000–2001, when they were voluntarily phased out by the manufacturers. However, use of these and other OP pesticides in agriculture continues, with approximately 73 million pounds applied in the United States each year [1]. Animal studies have found that prenatal exposure to various OP pesticides is associated with poorer fetal growth in rodents [2], [3], [4], [5], [6], [7], [8]. However, epidemiologic studies in humans have shown less consistency.

Whyatt et al. [9] found that concentrations of chlorpyrifos in umbilical cord blood were negatively associated with birth weight and length among infants born to low-income minority mothers in New York City before the year 2001. A recent small study of mothers and newborns in New Jersey found no association of chlorpyrifos in maternal or umbilical cord serum with any measure of fetal growth [10]; however, the second study was conducted after the residential phase-out and levels of chlorpyrifos exposure were considerably lower. A separate study carried out in New York City before the residential phase-out found no association of dialkyl phosphate (DAP) OP pesticide metabolites in maternal urine with birth weight or length, but did find a statistically significant inverse association with head circumference [11]. In a study of low-income women living in an agricultural community, we previously reported no association of maternal urinary DAP metabolites with decreased birth weight, length, or head circumference, but did find an association with shorter length of gestation [12].

One possible explanation for the lack of consistency in epidemiologic studies is individual and population-level differences in susceptibility to OPs [13], [14]. Paraoxonase 1 (PON1) is a high-density lipoprotein-associated enzyme whose primary physiological function appears to be related to metabolism of oxidized lipids [15], [16] and innate immunity [17], but which also plays a critical role in the metabolism and detoxification of OP pesticides. Animals with low PON1 activity are more sensitive to the toxic effects of some Ops, and treatment with exogenous PON1 can reduce an animal's response to the activated, oxon forms of OPs [18]. PON1's *in vitro* esterase activity can be measured against several substrates including paraoxon, chlorpyrifos-oxon, diazoxon, and phenyl acetate [19], [20].

There is a wide variability in PON1 enzyme activity in humans [21], which is influenced by several common single nucleotide polymorphisms (SNPs) in the *PON1* gene [22]. Of these, the most functionally important SNPs appear to be at position 192 in the coding region and −108 in the promoter region [23]. The *PON1_192_* SNP results in a Q/R amino acid substitution that affects the enzyme's catalytic efficiency, with the R alloform hydrolyzing chlorpyrifos-oxon more efficiently than the Q. In contrast, a T/C allelic substitution at position −108 in the promoter region impacts the amount of enzyme expressed, with the *PON1_−108CC_* genotype displaying significantly higher levels of plasma PON1 [24].

Few studies have examined the interaction of PON1 enzyme activity or genotype and OP pesticide exposure on birth outcome. In the same New York City population described above [11], Berkowitz et al. [25] found that urinary metabolites of chlorpyrifos were not associated with fetal growth on their own, but found significantly reduced head circumference in infants of mothers with both detectable levels of urinary metabolites of chlorpyrifos and low PON1 activity. Wolff et al. [11] also found statistically significant interaction between maternal PON1 genotype/activity and OP exposure for birth weight and length: maternal DAP metabolites were inversely associated with birth weight, but only among mothers with the more susceptible *PON1_192 QQ_* genotype, and inversely associated with birth length, but only among mothers with low paraoxonase activity [11]. Moreno-Banda et al. [26] examined the odds of low birth weight and found significant interaction between *PON1_192_* genotype and work in greenhouses among floriculture workers in Mexico; however, in this case, greenhouse work was associated with non-significantly increased odds of low birth weight among mothers with the *PON1_192 RR_* genotype.

The present study expands upon our previous analysis of maternal DAP metabolites and birth outcome, first by examining the main effect of PON1 on fetal growth and length of gestation and then by exploring interaction of maternal DAP levels and PON1.

## Methods

### Study participants

Participants were pregnant women enrolled in the Center for the Health Assessment of Mothers and Children of Salinas (CHAMACOS), a longitudinal cohort study investigating the effects of exposure to OP pesticides and other environmental chemicals on the health of pregnant women and their children [12]. The study population was comprised of low-income women living in the Salinas Valley, an agricultural region of northern California. Eligible women were at least 18 years of age, spoke English or Spanish, qualified for government-sponsored low-income health insurance (Medicaid), were less than 20 weeks gestation, and were receiving prenatal care at one of six clinics that served the farmworker community.

A total of 601 pregnant women were enrolled in the study and 538 were followed through delivery. We excluded stillbirths (n = 3), twins (n = 5), infants weighing <500 g at birth (n = 1), women with hypertension during pregnancy (n = 15), and women with existing or gestational diabetes (n = 26). We also excluded mother-infant pairs for whom we had no information on PON1 genotype or activity (n = 21), leaving a final sample size of 467. Of these, *PON1* genotype information was available for 451 mothers and 436 infants, and PON1 activity data were available for 371 mothers and 324 infants.

### Ethics Statement

We obtained signed informed consent from all women at the time of enrollment in the study. All study procedures were approved by the Committee for the Protection of Human Subjects at the University of California, Berkeley.

### Data Collection

Women were interviewed at the end of the first (mean ± standard deviation (SD) = 13.6±6.5 weeks) and second (mean ± SD = 25.8±2.5 weeks) trimesters of pregnancy. All interviews were conducted in English or Spanish by bilingual, bicultural interviewers using structured questionnaires.

Demographic data collected included maternal age, marital status, educational attainment, country of birth, number of years lived in the United States, language spoken in the home, family income, and the number of people supported by that income. Questionnaires also gathered information on behavioral characteristics, including tobacco, alcohol, drug, and caffeine use, and health information, including previous pregnancies and medical conditions. Body mass index (BMI) was calculated using self-reported pre-pregnancy weight and measured height. Data on pregnancy complications and birth outcomes, including infant birth weight, length, head circumference, and gestational age, were abstracted from medical records by a registered nurse.

### Pesticide Exposure Assessment

Spot urine samples were collected from mothers at the time of each interview and stored at −80°C until shipment to the Centers for Disease Control and Prevention (CDC) for analysis of OP pesticide metabolites. Dialkyl phosphate (DAP) metabolites were measured in urine samples using gas chromatography-tandem mass spectrometry (GC-MS/MS) and quantified using isotope dilution calibration [27]. Details of laboratory measurements and quality control are described elsewhere [28].

Six DAP metabolites were quantified: three dimethyl phosphate (DM) metabolites (dimethylphosphate, dimethyldithiophosphate, dimethylthiophosphate) which are derived from pesticides such as malathion, oxydemeton-methyl, and dimethoate, and three diethyl phosphate (DE) metabolites (diethylphosphate, diethyldithiophosphate, diethylthiophosphate) derived from pesticides such as diazinon, chlorpyrifos, and disulfoton. Although not all OP pesticides devolve to DAPs, approximately 80% of the OP pesticides used in the Salinas Valley devolve to one or more of these metabolites. DAP concentrations were converted to SI units (nanomoles per liter) and summed to generate variables for total DMs, total DEs, and total DAPs. All women had detectable levels of DAP metabolites in their urine. For a small number of women (n = 8) levels of one metabolite could not be calculated due to analytic interference. Because metabolites were highly correlated within the DE or DM groups, regression was used to impute the value of the missing metabolite based on the concentrations of the other two metabolites. Creatinine concentrations in urine were determined using a commercially available diagnostic enzyme method (Vitros CREA slides, Ortho Clinical Diagnostics, Raritan, NJ).

### PON1 Genotypes and Enzyme Activity

Maternal and fetal (umbilical cord) blood was collected at the time of delivery and stored at −80°C. Analysis of PON1 genotype and activity has been described previously [29]. Briefly, genotyping of the *PON1_−108_* and *PON1_192_* SNPs was performed using genomic DNA extracted from blood clots. For the coding SNP, *PON1_192_*, we used the Taqman real-time PCR method with probes custom-designed by Applied Biosystems, Inc. (Foster City, CA). For the promoter SNP, *PON1_−108_*, a two-part nested PCR strategy was used: first, the region surrounding the SNP was pre-amplified using non-allelic flanking primers, then the resulting amplicon was diluted and served as the template for the subsequent Amplifluor assay. Each plate of DNA incorporated randomly distributed blank samples and duplicate DNA samples independently isolated from the same subjects. We observed a high rate of concordance (>99%) among duplicate DNA samples (4% of samples). All discrepancies were resolved with additional genotyping.

Two substrate-specific PON1 enzyme activity assays (arylesterase and paraoxonase) were performed using spectrophotometric methods as described by Huen et al. [20]. Arylesterase activity, which measures the rate of hydrolysis of the substrate phenyl acetate (in µmol/min/L of plasma), reflects quantity of PON1 enzyme [30], [31]. In contrast, paroaxonase activity, which measures the rate of hydrolysis of paraoxon, reflects a combination of the catalytic efficiency and quantity of the PON1 enzyme. For quality assurance, repeat samples and internal controls were included in all assay runs. The average coefficient of variation (CV) for internal control samples repeated throughout different assay runs (inter-assay variability) was 7–9% and the average CV for repeated samples ranged from 6–9% [20]. Both substrate-specific enzymatic assays were performed in triplicate.

### Statistical Analyses

Initial analyses used linear regression to examine the main association of PON1 genotype and activity with length of gestation and infant birth weight, length, and head circumference independent of pesticide exposure. Maternal and child *PON1* genotypes were examined categorically; for *PON1_−108_*, the CC genotype is the reference, and for *PON1_192_*, the RR genotype is the reference. Arylesterase and paraoxonase activity in maternal and umbilical cord blood were examined as continuous variables, normalized by dividing by the standard deviation. Potential confounders were selected *a priori* from the set of characteristics known to be associated with birth outcome in this population. Covariates were kept in the final models if they were also associated with PON1 genotype or activity or if their exclusion from the model changed the coefficient on the main effect by more than 10%. Models of PON1 genotype included maternal pre-pregnancy BMI and maternal weight gain during pregnancy as covariates. Models of PON1 enzyme activity also included maternal age and country of birth, as well as assay temperature, since ambient temperature has been shown to affect hydrolysis rates [20]. To explore PON1 status, we examined the association of PON1 activity with birth outcome within each *PON1_192_* genotype by stratifying by genotype and by testing for interaction between activity and genotype using cross-product terms. As no statistically significant interaction was found, analyses of PON1 activity on birth outcome controlled for *PON1_192_* genotype as a covariate but not as an effect modifier. Models of birth weight, length and head circumference also controlled for gestational age and gestational age^2^ to examine associations independent of gestational duration.

Subsequent analyses explored whether PON1 genotype or activity was an effect modifier in the association of maternal DAP metabolite concentrations and length of gestation or fetal growth. Linear regression models were created for the association of total urinary concentrations of DMs, DEs and DAPs with these birth outcomes using the same methods and covariates as described in our previously published study [12], but including either PON1 genotype (categorical variable) or activity (continuous variable) and cross-product terms to test for interaction. For *PON1* genotype, two cross-product terms were included in the model (e.g. QR*DAP concentrations and RR*DAP concentrations), and the overall statistical significance of both terms in the model was determined using Wald tests. Similar cross-product terms were generated using tertiles of PON1 activity. Statistical significance for interaction was set at p-value<0.1. To further investigate effect modification by PON1, we then stratified the linear regression models by genotype or by tertiles of enzyme activity.

The variables for total concentrations of DMs, DEs and DAPs were log-transformed to reduce the impact of outlier points. The measurements at two time points during pregnancy were averaged to create a summary measure of *in utero* OP pesticide exposure. The analyses were conducted using DAP concentrations unadjusted for creatinine. Subsequent sensitivity analyses included creatinine as a covariate in the models.

Covariates included in the models were maternal age, pregnancy weight gain, gestational age at first prenatal care visit (in weeks), and gestational age at urine collection (in weeks) as continuous variables and parity (0 or ≥1), infant sex, country of birth (US or other), household income (≤poverty or >poverty), and maternal pre-pregnancy BMI (normal, overweight, or obese) as categorical variables. Smoking, alcohol, and drug use were not included in the final models because very few women reported use and their inclusion did not alter the results.

Statistical analyses were performed using STATA/IC version 10.1 (StataCorp LP, College Station, TX).

## Results

Characteristics of the study population are shown in Table 1. Women were predominantly Spanish-speaking and born in Mexico, with 41% of women working in agriculture during pregnancy. More than 60% of families were living below the federal poverty threshold and 89% of women had never completed high school. The mean age was 25.5 years (SD = 5.0). All women had detectable levels of OP pesticide metabolites in their urine during pregnancy. The geometric mean (GM) for the average DAP concentrations during pregnancy was 146 nmol/L (95% confidence interval (CI) = 133, 160); of this, a larger proportion was DM metabolites (GM = 109 nmol/L; 95% CI = 98, 120) than DEs (GM = 23 nmol/L; 95% CI = 21, 25).

**Table 1:** Demographic characteristics of CHAMACOS study population, Salinas Valley, California, 2000–2001 (N = 467).

Characteristic	N (%)
Age (years)	
18–24	225 (48.2)
25–29	144 (30.8)
30–34	69 (14.8)
≥35	29 (6.2)
Parity	
0	155 (33.2)
≥1	312 (66.8)
Marital status	
Single	94 (20.1)
Married/Living as Married	373 (79.9)
Language spoken at home	
Spanish	412 (88.2)
English	28 (6.0)
Both Spanish and English	23 (4.9)
Other	4 (0.9)
Country of birth	
Mexico	393 (84.2)
United States	64 (13.7)
Other	10 (2.1)
Family Income	
Below poverty threshold	288 (61.7)
Above poverty threshold	179 (38.3)
Education	
≤6th grade	198 (42.4)
7–12th grade	170 (36.4)
High School Graduate	99 (21.2)
Pre-pregnancy BMI	
Underweight	3 (0.7)
Normal	174 (38.2)
Overweight	183 (40.2)
Obese	95 (21.0)
Smoking during pregnancy	
No	438 (93.8)
Yes	29 (6.2)
Work status during pregnancy	
Worked in agriculture	188 (41.1)
Worked in other industry	104 (22.8)
Did not work	165 (36.1)
Infant sex	
Boy	234 (50.1)
Girl	233 (49.9)

The allele frequencies of the *PON1_192_* Q allele and the *PON1_−108_* T allele were 50% and 46% in this population, respectively, and did not differ between mothers and newborns. The mean arylesterase and paraoxonase activity was 33.6 U/mL (SD = 16, range = 4–145) and 256.6 U/L (SD = 165, range = 7–1018), respectively, for infants and 136.6 U/mL (SD = 44, range = 27–347) and 989.0 U/L (SD = 616, range = 75–3538) for mothers.

Table 2 shows that individuals' arylesterase and paraoxonase activity varied by genotype. As expected, arylesterase activity (a marker of enzyme quantity) was lowest among mothers and infants with the *PON1_−108TT_* genotype and paraoxonase activity (a combined measure of both quantity and enzyme efficiency) was lower among mothers and infants with either the *PON1_−108TT_* or *PON1_192QQ_* genotype, suggesting that individuals with these genotypes might be more susceptible to the adverse effects of OP pesticide exposure. The lowest arylesterase and paraoxonase levels were seen in mothers and infants who were homozygous for both *PON1_−108TT_* and *PON1_192QQ_*.

**Table 2:** Distribution of PON1 genotype and enzyme activity in mothers and neonates, CHAMACOS Study, Salinas Valley, CA.

			Arylesterase Activity (U/mL)			Paraoxonase Activity (U/L)		
		N	Mean (SD)			Mean (SD)		
Maternal genotype								
*PON1_−108_*	CC	106	149.83	(45.87)	a	1346.00	(694.66)	b
	CT	171	136.22	(41.23)	a	938.95	(536.77)	b
	TT	88	119.02	(40.24)	a	676.48	(444.31)	b
*PON1_192_*	RR	95	130.06	(38.70)	c	1659.00	(561.49)	d
	QR	170	134.28	(39.39)		997.65	(341.46)	d
	QQ	101	145.19	(53.38)	c	356.68	(269.28)	d
*PON1_−108_*×*PON1_192_*	CC/RR	44	35.44	(11.57)		338.17	(160.44)	
	CC/QR	41	40.65	(18.27)		299.52	(183.40)	
	CT/RR	30	35.55	(20.91)		328.17	(198.82)	
	CT/QR	73	34.38	(14.87)		276.03	(148.02)	
	CC/QQ	14	32.37	(10.25)		168.59	(138.74)	*
	TT/RR	13	31.59	(11.78)		305.90	(155.16)	
	TT/QR	34	25.50	(12.03)		173.16	(117.64)	*
	CT/QQ	41	30.54	(14.94)		178.25	(142.59)	*
	TT/QQ	30	31.86	(24.56)		170.37	(108.54)	*
Infant genotype								
*PON1_−108_*	CC	94	41.78	(12.98)	e,f	343.00	(169.01)	g
	CT	167	31.75	(13.76)	e	238.38	(145.43)	g
	TT	57	26.52	(22.76)	f	168.53	(147.87)	g
*PON1_192_*	RR	76	36.60	(13.18)		405.68	(170.90)	h
	QR	172	33.62	(18.06)		258.72	(117.67)	h
	QQ	75	30.54	(15.04)		100.84	(96.13)	h
*PON1_−108_*×*PON1_192_*	CC/RR	34	43.47	(10.81)		481.06	(154.03)	
	CC/QR	47	40.61	(13.54)		296.87	(103.78)	*
	CT/RR	33	30.39	(12.43)	*	347.72	(169.60)	*
	CT/QR	92	32.15	(14.40)	*	261.78	(103.70)	*
	CC/QQ	13	41.57	(16.38)		148.68	(107.73)	*
	TT/RR	8	32.79	(13.20)		309.70	(116.79)	*
	TT/QR	30	28.96	(28.94)	*	202.71	(149.12)	*
	CT/QQ	42	31.92	(13.56)	*	101.22	(95.87)	*
	TT/QQ	19	20.03	(11.10)	*	55.14	(50.21)	*

a,b,c,… Values with the same superscript are significantly different (p<0.05) in pairwise Bonferroni's multiple comparison tests (*PON1_−108_* and *PON1_192_* analyses).

*Values significantly different (p<0.05) from CC/RR individuals in pairwise Bonferroni's multiple comparison tests (*PON1_−108_*×*PON1_192_* analyses).

The independent association of infant PON1 genotype or enzyme activity with birth outcome, without consideration of pesticide metabolite levels, is shown in Table 3. Separate regression models are shown for each genotype; the model for arylesterase also includes *PON1_192_* genotype to better capture overall PON1 status. On average, infants with the susceptible *PON1_−108TT_* genotype were born earlier (β = −0.5 weeks, 95% CI: −0.9, 0.0) and had smaller head circumferences (β = 0.4 cm, 95% CI: −0.7, 0.0), after controlling for confounders, than infants with the *PON1_−108CC_* genotype. Smaller head circumference was also seen among infants with the *PON1_−108CT_* genotype (β = −0.3 cm, 95% CI: −0.6, 0.0) compared to those with *PON1_−108CC_*. No associations were seen with *PON1_192_*; however, when the *PON1_−108_* and *PON1_192_* genotypes were combined, we observed shorter gestation (β = −1.1 weeks, 95% CI: −1.9, −0.4), birth weight (β = −184.0 g, 95% CI: −372.4, 4.4), and head circumference (β = −0.8 cm, 95% CI: −1.4, −0.2) among infants with double homozygote *PON1_−108TT/192QQ_* genotypes compared to those with *PON1_−108CC/192RR_*, with the magnitude of effect considerably larger than for *PON1_−108_* genotype alone.

**Table 3:** Association of infant PON1 genotype (N = 436) and activity (N = 324) with birth outcome, CHAMACOS Study, Salinas Valley, CA.

			Gestational Age (weeks)		Birth Weight^a^ (g)		Head Circumference^a^ (cm)	
		N	β	( 95% CI )	β	( 95% CI )	β	( 95% CI )
**Infant Genotype**								
***PON1_−108_***	**CC**	131	ref		ref		ref	
	**CT**	225	0.1	(−0.3, 0.4)	−67.8	(156.2, 20.7)	−0.3	(−0.6, 0.0)*
	**TT**	76	−0.5	(−0.9, 0.0)*	−36.0	(−152.3, 80.3)	−0.4	(−0.7, 0.0)
***PON1_192_***	**RR**	106	ref		ref		ref	
	**QR**	222	0.1	(−0.3, 0.4)	−19.8	(−113.5, 73.9)	−0.1	(−0.4, 0.2)
	**QQ**	108	−0.3	(−0.7, 0.2)	−73.9	(−183.8, 36.0)	−0.2	(−0.6, 0.1)
***PON1_−108_*** **×** ***PON1_192_***								
	**CCRR**	49	ref		ref		ref	
	**CCQR**	57	−0.1	(−0.7, 0.5)	−51.8	(−207.7, 104.2)	−0.2	(−0.7, 0.3)
	**CTRR**	48	−0.2	(−0.9, 0.4)	−140.7	(−303.4, 21.9)	−0.5	(−1.1, 0.0)
	**CTQR**	123	−0.1	(−0.5, 0.6)	−128.8	(−263.3, 5.7)	−0.5	(−1.0, −0.1)*
	**CCQQ**	25	−0.3	(−1.1, 0.5)	−163.0	(−358.8, 32.8)	−0.5	(−1.1, 0.2)
	**TTRR**	8	−0.2	(−1.4, 1.0)	−47.7	(−350.4, 254.9)	−0.8	(−1.8, 0.1)
	**TTQR**	39	−0.2	(−0.9, 0.4)	−30.1	(−200.9, 140.6)	−0.3	(−0.8, 0.3)
	**CTQQ**	53	0.0	(−0.7, 0.6)	−105.6	(−263.9, 52.8)	−0.4	(−0.9, 0.1)
	**TTQQ**	29	−1.1	(−1.9, −0.4)*	−184.0	(−372.4, 4.4)	−0.8	(−1.4, −0.2)*
**Infant PON1 Status**								
**Arylesterase** b		322	0.2	(0.1, 0.4)*	34.6	(−10.9, 80.1)	0.1	(−0.1, 0.2)

*p-value<0.05.

a Models adjusted for maternal BMI, maternal weight gain, gestational age, and (gestational age)^2^.

b Models additionally adjusted for *PON1_192_* genotype, maternal age, country of birth, and assay temperature. Change per 1 standard deviation increase in activity.

Similar results were seen with infant PON1 enzyme activity. After controlling for *PON1_192_* genotype, each standard deviation increase in arylesterase activity was associated with 0.2 week (95% CI: 0.1, 0.4) increase in gestational duration (Table 3). Paraoxonase activity was similarly positively associated with gestational age (not shown). Neither arylesterase nor paraoxonase activity was associated with birth weight, length, or head circumference after controlling for confounding variables.

The associations of PON1 with birth outcome were found only with PON1 measured in infants, not in mothers. No associations were found between any marker of PON1 genotype or activity measured in maternal blood and gestational age or infant birth weight, length, and head circumference (Table S1).

**Figure 1:**
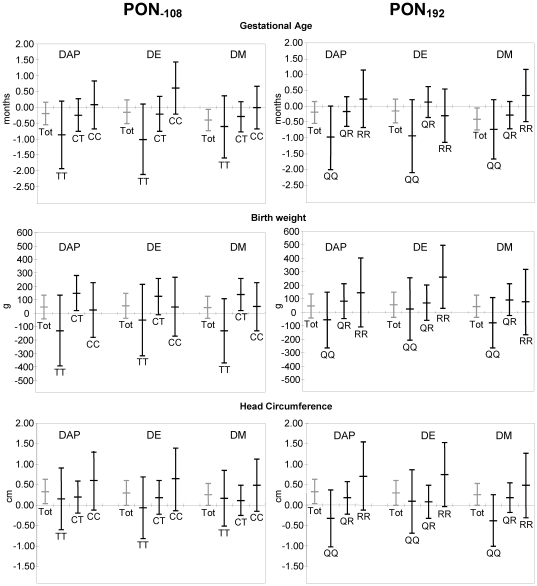
Association of maternal urinary DAPs with birth outcome, stratified by infant PON1 genotype. Models of gestational age controlling for timing of urine collection, timing of entry into prenatal care, maternal age, parity, country of birth, and poverty level. Models of birth weight and head circumference control for timing of urine collection, timing of entry into prenatal care, maternal age, parity, infant sex, country of birth, weight gain, BMI, poverty level, gestational age, and (gestational age)^2^.

Results of the analysis of interaction between DAPs, PON1 and birth outcome are shown in Table 4 along with the adjusted association of DAPs and birth outcomes, stratified by either PON1 genotype or tertiles of arylesterase activity. The data stratified by genotype are also shown graphically in Figure 1. Our previously reported [12] associations of DAP concentrations in maternal urine and birth outcome, not accounting for PON1, are shown in grey on Figure 1. We previously reported a decrease in length of gestation with prenatal DM metabolite levels and an unexplained pattern of increased fetal growth, particularly for head circumference, with DAP metabolite levels. In stratified analyses, maternal DAP, DM and DE metabolite levels were all negatively associated with length of gestation among infants with the susceptible *PON1_−108TT_* and *PON1_192QQ_* genotypes. The magnitude of the association was consistently larger in the susceptible children than in the total population, but did not reach statistical significance, likely due to the reduced sample sizes. Each ten-fold increase in maternal DAPs during pregnancy was associated with a 0.9 week (95% CI: −2.0, 0.2) decrease in gestational age among *PON1_−108TT_* infants and a 1.0 week (95% CI: −2.0, 0.0) decrease among *PON1_192QQ_* infants (Table 4 and Figure 1).

**Table 4:** Adjusted association of log10 increase in DAPs and birth outcome, stratified by child PON1 genotype or tertiles of arylesterase activity, CHAMACOS Study, Salinas Valley, CA.

	Gestational Age^a^				Birth Weight^b^			Head Circumference^b^		
	N	β	(95% CI)	Interaction P-value	β	(95% CI)	Interaction P-value	β	(95% CI)	Interaction P-value
**PON1_−108_**										
**Total DAPs**										
**TT**	76	−0.8	(−2.0, 0.2)	0.36	−131.3	(−393.3, 130.8)	0.06 †	0.1	(−0.6, 0.9)	0.08 †
**CT**	225	−0.3	(−0.8, 0.3)		147.2	(18.5, 275.7)*		0.2	(−0.2, 0.6)	
**CC**	131	0.1	(−0.7, 0.8)		22.1	(−182.0, 226.3)		0.6	(−0.1, 1.3)	
**DEs**										
**TT**	76	−1.0	(−2.1, 0.1)	0.09 †	−55.3	(−320.5, 209.8)	0.35	−0.1	(−0.8, 0.7)	0.19
**CT**	225	−0.2	(−0.8, 0.3)		120.8	(−14.8, 256.4)		0.2	(−0.2, 0.6)	
**CC**	131	0.6	(−0.2, 1.4)		45.4	(−174.6, 265.4)		0.6	(−0.2, 1.4)	
**DMs**										
**TT**	76	−0.6	(−1.6, 0.4)	0.49	−135.2	(−373.8, 103.3)	0.05 †	0.2	(−0.5, 0.8)	0.12
**CT**	225	−0.3	(−0.8, 0.2)		134.6	(14.9, 254.2)*		0.1	(−0.3, 0.5)	
**CC**	131	0.0	(−0.7, 0.7)		46.8	(−132.5, 226.1)		0.5	(−0.2, 1.1)	
**PON1_192_**										
**Total DAPs**										
**QQ**	108	−0.1	(−2.0, 0.0)	0.21	−60.2	(−266.3, 145.9)	0.20	−0.3	(−1.0, 0.4)	0.01 †
**QR**	222	−0.2	(−0.7, 0.3)		79.4	(−48.5, 207.3)		0.2	(−0.2, 0.6)	
**RR**	106	0.2	(−0.7, 1.1)		142.3	(−114.6, 399.3)		0.7	(−0.1, 1.5)	
**DEs**										
**QQ**	108	−1.0	(−2.1, 0.2)	0.17	20.5	(−210.5, 251.5)	0.30	0.1	(−0.7, 0.9)	0.27
**QR**	222	0.1	(−0.4, 0.6)		67.2	(−63.3, 197.5)		0.1	(−0.3, 0.5)	
**RR**	106	−0.3	(−1.2, 0.6)		258.8	(23.9, 493.6)*		0.7	(0.0, 1.5)	
**DMs**										
**QQ**	108	−0.7	(−1.7, 0.2)	0.25	−80.6	(−269.2, 107.9)	0.16	−0.4	(−1.0, 0.2)	0.01 †
**QR**	222	−0.3	(−0.7, 0.1)		89.9	(−27.8, 207.5)		0.2	(−0.2, 0.5)	
**RR**	106	0.3	(−0.5, 1.2)		72.9	(−169.2, 315.0)		0.5	(−0.3, 1.3)	
**Arylesterase Activity**										
**Total DAPs**										
**Low**	108	0.3	(−0.5, 1.2)	0.17	−14.6	(−263.4, 234.3)	0.39	−0.2	(−1.0, 0.5)	0.32
**Middle**	108	−0.4	(−1.2, 0.4)		63.8	(−131.1, 258.7)		0.3	(−0.3, 0.9)	
**High**	108	−0.2	(−0.9, 0.5)		92.2	(−106.6, 291.1)		0.8	(0.1, 1.4)*	
**DEs**										
**Low**	108	−0.	(−1.2, 0.4)	0.69	−55.2	(−295.6, 185.2)	0.31	−0.5	(−1.2, 0.2)	0.48
**Middle**	108	−0.7	(−1.5, 0.1)		48.7	(−134.4, 231.8)		0.4	(−0.2, 1.0)	
**High**	108	0.5	(−0.3, 1.2)		231.4	(19.1, 443.6)		0.7	(−0.1, 1.4)	
**DMs**										
**Low**	108	0.5	(−0.3, 1.2)	0.16	14.8	(−216.2, 245.7)	0.29	−0.1	(−0.8, 0.5)	0.36
**Middle**	108	−0.4	(−1.1, 0.4)		83.9	(−94.8, 262.6)		0.1	(−0.4, 0.7)	
**High**	108	−0.4	(−1.0, 0.3)		60.2	(−120.2, 240.7)		0.7	(0.1, 1.3)*	

a Gestational age models adjusted for timing of urine collection, timing of entry into prenatal care, maternal age, parity, country of birth, and household income.

b Models of birth weight and head circumference adjusted for timing of urine collection, timing of entry into prenatal care, maternal age, parity, country of birth, household income, prepregnancy BMI, maternal weight gain, infant sex, gestational age, and (gestational age)^2^.

*p-value on stratified association <0.05.

† p-value for interaction <0.1.

Figure 1 shows that the positive associations that we reported between DAP concentrations and birth weight and head circumference (in grey) persisted among infants with the non-susceptible PON1 profiles. For example, among infants with the *PON1_192RR_* genotype, each ten-fold increase in prenatal DEs was associated with a 258.8 g (95% CI: 23.9, 493.6) increase in birth weight and a 0.7 cm (95% CI: 0.0, 1.5) increase in head circumference. Each 10-fold increase in DMs was associated with a 0.8 cm (95% CI: 0.1, 1.4) increase in head circumference among infants with high levels of arylesterase activity (Table 4). However, among the susceptible infants with *PON1_−108TT_* or *PON1_192QQ_* genotype or low arylesterase activity, DAPs, DEs and DMs were either not associated with fetal growth or were trending towards decreased growth.

Results of tests for interaction between PON1 and DAPs are shown in Table 4. Interaction p-values were derived from the full regression models that included DAPs, genotype or tertiles of activity, and cross-product terms, and represent the overall p-value on both cross-product terms using the Wald test. Statistically significant interaction was seen between DAPs (specifically DMs) and PON1 genotype for head circumference (p-value_interaction terms_ = 0.08 for *PON1_−108_* and 0.01 for *PON1_192_*) and birth weight (p-value_interaction terms_ = 0.06 for *PON1_−108_*). This interaction appears to be driven by positive associations of DAPs with fetal growth among those with non-susceptible genotypes. We did find statistically significant interaction between DE metabolites and *PON1_−108_* genotype for gestational age, supporting our finding that prenatal DE exposure was associated with shorter length of gestation only among the susceptible *PON1_−108TT_* infants. Although we observed similar patterns with gestational age for *PON1_192_* and for DAPs and DMs, the interaction terms for these models were not statistically significant.

## Discussion

This study found that infant, but not maternal, PON1 genotype and activity were associated with birth outcome in a population of pregnant women living in an agricultural community. Specifically, we observed shorter gestation and smaller head circumference among infants who might be more susceptible to the effects of exposure to OP pesticides, either because they had lower PON1 enzyme activity (measured as arylesterase or paraoxonase activity) or because they were of a susceptible genotype (*PON1_−108TT_*). This suggests that low PON1 activity may be a factor contributing to preterm birth or restricted fetal growth.

It is not clear whether this association of PON1 and birth outcome is mediated through PON1's role in OP pesticide metabolism or oxidative stress. However, we did find evidence that PON1 may be an effect modifier in the association of OP pesticide exposure with birth outcome. Maternal DAP, DE, and DM metabolite concentrations were associated with shorter duration of gestation among infants with the susceptible *PON1_−108TT_* and *PON1_192QQ_* genotypes, although these findings were not statistically significant within individual strata. Although the patterns of decreased gestational age with DAP concentrations were similar across both susceptible genotypes and for all types of DAP metabolites, only the interaction between *PON1_−108_* genotype and DE metabolite concentrations was statistically significant.

Our finding of an independent effect of PON1 genotype and activity on birth outcome is consistent with the New York cohort studied by Berkowitz et al. [25] and Wolff et al [11], the only other study to examine *PON1_−108_* and arylesterase with birth outcome (see Table 5). Like us, they found *PON1_−108TT_* genotype to be associated with smaller infant head circumference [25]. They also found lower arylesterase activity to be associated with smaller head size, while we found it to be associated with shorter gestation. A difference in the studies is that, although both looked at maternal and infant PON1, all of their associations were with maternal PON1 genotype and activity while ours were with infant PON1.

**Table 5:** Comparison of studies of PON1 and birth outcome.

Study	Population	*PON1_−108_* 1		*PON1_192_* 1		Arylesterase^2^		Paraoxonase^2^		Interaction with OP pesticides?
		Infant	Mother	Infant	Mother	Infant	Mother	Infant	Mother	
Harley, 2010	N = 470Rural California	TT:↓ GA,↓ Head	Null	Null	Null	+GA	Null	+GA	Null	Among TT infants:↓ GA with DEsAmong CT or CC infants↑ Head, ↑ BW with DAPs, DMsAmong RR infants↑ Head with DAPs, DMs
Berkowitz, 2004	N = 404New York City	Null	TT:↓ Head	–	–	Null	+Head	–	–	Among mothers with low arylesterase:↓ Head with Chlorpyrifos
Chen, 2008	N = 185China	–	–	RR: Increased preterm	Null	–	–	–	–	–
Lawlor, 2006	N = 4286UK	–	–	–	RR:Increased preterm	–	–	–	–	–
Min, 2006	N = 276Korea	–	–	–	RR:↓ BW	–	–	–	–	–
Moreno-Banda, 2009	N = 264Mexico	–	–	–	Null	–	–	–	–	Among QQ mothers:↓ LBW with work in floriculture
Roy, 1994	N = 91Singapore	–	–	–	–	–	–	–	−BW	–
Ryckman, 2010	N = 424NorwayN = 764Tennessee	–	–	Null	Null	–	–	–	–	–
Wolff, 2007	N = 404New York City	–	–	Null	RR:↓ BW↓ Head	Null	+Head	–	–	Among QQ mothers:↓ BW with DEsAmong mothers with low arylesterase:↓ length with DMs

Abbreviations: BW = birth weight, LBW = low birth weight, GA = gestational age, Head = head circumference, – not examined.

1 ↓GA indicates that genotype is associated with decreased GA; ↑GA indicates association with increased GA.

2 +GA indicates that enzyme activity is positively associated with GA; −GA indicates negative association with GA.

Results with *PON_192_* and paraoxonase activity are less consistent (Table 5). Only one small study has examined paraoxonase activity and birth weight; Roy et al.found the opposite of our study, reporting that higher maternal paraoxonase activity was associated with lower birth weight [32]. More studies have examined *PON1_192_* [11], [33], [34], [35]. Consistent with our study, Ryckman et al. [36] reported no association of the *PON1_192_* maternal or child genotype with preterm birth in two cohorts in Norway or Tennessee. However, other studies found that mothers or infants with the *PON1_192RR_* genotype were at increased risk of preterm birth [33], [34] shorter gestational age [35], or smaller birth size [11]. These findings with *PON1_192_* are the opposite of our hypothesis that individuals with the *PON1_192RR_* would be at *lower* risk of poor birth outcomes because of their improved efficiency at detoxifying OP pesticides. However, individuals with the *PON1_192RR_* genotype appear to be *more* susceptible to certain oxidative stress pathways, another important mechanism for the PON1 enzyme. Another possible explanation for the associations of *PON1_192RR_* with poorer birth outcome found in these studies is uncontrolled confounding by *PON1_−108_*, since the two SNPs are in linkage disequilibrium (D′ = 0.22, p-value 0.01) [29].

Our evidence for interaction between PON1 and OP exposure on birth outcome is supported by three other studies [11], [25], [26]. None of these studies examined length of gestation, where we see the strongest result in this study. All three previous studies report statistically significant interaction between PON1 and markers of pesticide exposure on fetal growth, although the details of this interaction differ (Table 5). In the New York population, DE metabolite concentrations were negatively associated with birth weight in the *PON1_192QQ_* mothers [11]. We find DE concentrations to be negatively associated with length of gestation in the *PON1_192QQ_* and *PON1_−108TT_* infants. The New York studies also found that, among mothers with low arylesterase levels, DM concentrations were negatively associated with birth length [11] and levels of a urinary metabolite of chlorpyrifos (a DE pesticide) were negatively associated with head circumference [25]. Thus, although there are differences in outcomes and in whether the effect modification is due to infant or maternal PON1, these studies all suggest that some individuals may be more susceptible to negative health effects of resulting from exposure to OP pesticides.

Examining interaction by PON1 expands on our earlier findings of shorter duration of gestation with increasing *in utero* DM levels. However, the only significant interaction that we observed for gestational age was with DE concentrations, not DMs. It is possible that the reason we did not observe an association of gestational age with DE metabolites previously is that it was masked by the role of PON1. Observing interaction with DE concentrations is plausible since the known biologically relevant action of PON1 is with two DE pesticides, chlorpyrifos and diazinon. PON1 does not hydrolyze DM pesticides such as malathion, oxydemeton-methyl, and dimethoate directly, but may have an indirect effect on their toxicity; by detoxifying chlorpyrifos oxon or diazoxon, PON1 prevents these OPs from inhibiting the carboxylesterases that detoxify some of the DM OPs. Thus, interaction of PON1 and DM metabolites is not unexpected.

We previously reported borderline increases in birth weight and head circumference associated with *in utero* DAP concentrations. These findings are in contrast with other studies, notably Whyatt et al., who that found OP pesticide levels in maternal blood were associated with decreased fetal growth. In fact, stratification by PON1 status served to magnify some of those positive associations, such that in the non-susceptible groups (e.g. *PON1_−108CC_*, *PON1_192RR_*, and high arylesterase), increasing DAP concentrations were associated with increased birth weight and head circumference. We have no ready explanation for these findings, although it is possible that, among the less susceptible groups, high DAP concentrations in urine are actually an indication of high metabolizers (i.e. those who detoxify and excrete OP pesticides quickly), rather than high exposure.

The use of DAP metabolites to measure exposure has some limitations. DAPs measure short term exposure to OP pesticides. Although we took the average of two DAP measurements, one in early and one in late pregnancy, they may not reflect on-going exposure throughout gestation. Additionally, DAP metabolites in urine may overestimate OP pesticide exposure to the extent that they reflect exposure to preformed DAPs in the environment as well as to the parent OP compound [37]. Also, it is not known how DAP metabolite levels and excretion patterns may differ by PON1 status, even among individuals with similar exposures.

Despite its large sample size, this study also lacked power to thoroughly examine interaction and conduct stratified analyses. We examined only two main polymorphisms, *PON1_192_* and *PON1_−108_*. Although we have recently shown that other PON1 polymorphisms are also associated with arylesterase and paraoxonase activity in this cohort, most of them were in strong linkage disequilibrium with the two examined in this study [38].

In summary, we found evidence for a main effect of infant *PON1_−108TT_* genotype and low arylesterase and paraoxonase activity on shorter gestation and smaller head circumference. This finding has important implications as a potential risk factor for preterm birth and fetal growth restriction and adds to the growing body of evidence that PON1 genotype may affect child development [39]. In addition to its role in detoxification and metabolism of OP pesticides, PON1 appears to also play a strong role in metabolism of oxidized lipids which was beyond the scope of this study.

This study also provides additional evidence that exposure to OP pesticides may impact fetal growth and length of gestation, particularly among individuals with increased susceptibility to OP pesticides. Additional studies are needed to help resolve continuing inconsistencies between the small number of existing studies.

## Supporting Information

Table S1 **Association of maternal PON1 genotype (N = 451) and activity (N = 371) with birth outcome, CHAMACOS Study, Salinas Valley, CA.**
(DOC)
