# Supplementary material for: Association of Organophosphate Pesticide Exposure and Paraoxonase with Birth Outcome in Mexican-American Women
Source: PLoS One. 2011 Aug 31;6(8):e23923. doi: 10.1371/journal.pone.0023923 (PMC3164135; doi:10.1371/journal.pone.0023923)
Supplement: Table S1 — Association of maternal PON1 genotype (N = 451) and activity (N = 371) with birth outcome, CHAMACOS Study, Salinas Valley, CA. (DOC) [file pone.0023923.s001.doc]

Supplemental Table 1: Association of maternal PON1 genotype (N=451) and activity (N=371) with birth outcome, CHAMACOS Study, Salinas Valley, CA.

**Gestational Age (weeks)   Birth Weight^a^ (g) Head Circumference^a^ (cm)**

**N β ( 95% CI )**   **β ( 95% CI ) β ( 95% CI )**

**Maternal Genotype**

***PON1_-108_* CC** 127 ref ref ref

**CT** 219 -0.03 (-0.36, 0.30) 11.67 (-82.19, 105.52) 0.01 (-0.29, 0.32)

**TT** 105 0.01 (-0.39, 0.40) -50.68 (-161.58, 60.22) -0.27 (-0.63, 0.09)

***PON1_192_***

**RR** 118 ref ref ref

**QR** 207 -0.10 (-0.45, 0.24) 72.11 (-24.51, 168.73) -0.01 (-0.33, 0.30)

**QQ** 126 -0.06 (-0.44, 0.33) 48.70 (-58.73, 156.13) 0.13 (-0.22, 0.48)

**Maternal PON1 activity**

**ARYase^b^** 371 0.00 (-0.16, 0.15) -20.93 (-64.98, 23.11) 0.02 (-0.12, 0.16)

**POase^b^** 371 -0.02 (-0.17, 0.13) -9.42 (-53.09, 34.26) 0.00 (-0.14, 0.14)

* p-value < 0.05

^a^ Models adjusted for gestational age and (gestational age)^2

^b^ Models additionally adjusted for assay temperature. Change per 1 standard deviation increase in activity.
